# Advancing Physical Therapy Practice through Curriculum Revision: The Malawi Experience

**DOI:** 10.3389/fpubh.2017.00216

**Published:** 2017-08-18

**Authors:** Janna Beling, Enock Chisati

**Affiliations:** ^1^Department of Physical Therapy, California State University, Northridge, Northridge, CA, United States; ^2^Physiotherapy Department, College of Medicine, University of Malawi, Blantyre, Malawi

**Keywords:** physiotherapy, curriculum, health education reform, instruction, pedagogy, Africa

## Abstract

Challenged health systems are a motivation for health education reform. Although resources-limited areas cover our planet, sub-Saharan Africa has the highest disease burden, yet the lowest health-care provider and medical school density of any region in the world. Malawi is among the most under-resourced countries in the world. While much of the data focus on dental, medical, and psychiatric service provision, physical therapists are also in short supply. Among the barriers to achieving the recommended standards for physical therapist education, African physiotherapists (the term for “physical therapists” in Africa) identify limited training opportunities, limited research education, and limited resources and funding. The purpose of this article is to describe an international partnership for strengthening the Malawian physiotherapist workforce capacity through curriculum revision in the Department of Physiotherapy at the University of Malawi’s College of Medicine.

## Background

Malawi is a small country in southeast Africa with a population of 17,215,000 (1) and an acute shortage of rehabilitation workers: a ratio of 0.8 physiotherapists per 100,000 people (by comparison the United States has nearly 1,350 per 100,000 people) (2). Malawi has 147 qualified physiotherapists and 27 physiotherapist interns registered with the Medical Council of Malawi (3). This number overstates the Malawian workforce because it includes physiotherapists who are volunteers during the year and are not residing in Malawi. At the time of this evaluation, only 26 physiotherapists had been trained in Malawi; therefore, more than three-quarters of the physiotherapists had been trained outside of the country. Historically, Malawi has depended on expatriates in the provision of these services through projects like Malawi against Physical Disabilities and the Sue Ryder Foundation (4).

Physiotherapists are represented worldwide by the World Confederation for Physical Therapy (WCPT) (5). According to the WCPT, physiotherapy is a vital portion of the health-care delivery system concerned with maximizing musculoskeletal function, within the areas of disability prevention, health promotion, rehabilitation, and treatment. Physiotherapists provide services to clients to develop, preserve, and return functional capacity and maximal movement throughout the lifecycle. It is at the core of impairment and disability prevention (5).

The provision of physiotherapy services in Malawi has lagged far behind other health services in the country. In Malawi, physiotherapy activities started back in the mid-sixties in response to polio outbreaks and only modest improvements have been made since that time (4). Despite the dearth of physiotherapists, the demand for rehabilitation services has continued to rise due to high numbers of road traffic accidents, diseases of lifestyle, HIV, and AIDS, and more people seeking high standards of health care which includes functional restoration (6). Malawi’s HIV prevalence is one of the highest in the world, with 14.5% of the population living with HIV in the most prevalent region of southern Malawi (7), where the College of Medicine (COM) is located. People with HIV infection often develop multiple complications requiring the need for physiotherapy such as tuberculosis of the spine, neuropathies, and meningitis (8). Workforce needs are growing for physiotherapists around the world. Malawi’s population is expected to triple by 2050 (1). A growing population will only increase the need for rehabilitation professionals in Malawi as a significant portion of the population is under 18 years of age and as it ages, the incidence of chronic disease will increase (9).

## Physiotherapy Education in Malawi

### Development of the Physiotherapy Program at the University of Malawi

A 1998 Ministry of Health Unit Review revealed that the Ministry had no policy on medical rehabilitation services; there was no staff development plan for rehabilitation personnel and very few facilities where people could access physiotherapy services in the country (10). The review, therefore, recommended that the Ministry should support the establishment of a school of physiotherapy in Malawi. The recognition of the shortage of rehabilitation workers was instrumental in approving, funding, and enrolling students in a new Department of Physiotherapy, which was started in January 2010 at the University of Malawi’s COM in Blantyre. The department offers a Bachelor of Science (B.Sc.) Honours in Physiotherapy degree. After students complete their degree, they are expected to complete a 1-year internship. The first two cohorts of 46 Malawian physiotherapists graduated from the COM in 2014 and 2015.

Before 2010, Malawi had no program to train physiotherapists within the country. All Malawian physiotherapists since 1968 were trained in the United Kingdom, Germany, South Africa, Zambia, Zimbabwe, Kenya, and Tanzania. This mode of training failed to produce significant numbers of physiotherapists to meet the needs of Malawi because of the prohibitive costs of and lack of access to foreign training. The development of the B.Sc. program at the University of Malawi’s COM was a significant step in producing locally trained physiotherapists to meet the country’s needs.

The WCPT defines physical therapy as a dynamic health-care profession that aims to assist individuals with the achievement, maintenance, and restoration of maximal physical functioning and health throughout their life (5). Not only is physiotherapy intervention associated with improvements in physical function, but in health-related quality of life (11). Physiotherapists have a leadership role in the prevention of loss of function and maintenance of functional mobility for individuals within communities. This is a very important role for physiotherapists and is endorsed by the WCPT (5). Physiotherapists can use their extensive knowledge and skills to promote improved public health by being involved in programs that promote health and wellness and reduce the risk of injury (12). People with a disability have a lower standard of living in Malawi and less than 25% of people with disabilities receive rehabilitation (13). Approximately 80% of people in Malawi live in rural areas (14) and rural people depend mostly on the physical demands of farming (15). Timely access to physiotherapy is not only beneficial to the patient, but, to society as a whole in order to insure functional mobility and economic stability.

### Capacity of the COM to Host the B.Sc. Physiotherapy Degree

The mission statement of the COM includes the training of cadres in the health sector in addition to medical doctors. It is responsible for developing high quality, entry-level programs in the health sciences that will produce graduates who can meet the service demands of the people of Malawi and its neighbors in the southern African region. The COM has committed senior management and academic staff and has established good links with the Ministry of Health for the implementation of the Physiotherapy program. The College has successfully sought funds to build its own lecture hall and other purpose-built physiotherapy teaching areas as well as an outpatient faculty physiotherapy clinic. The Physiotherapy Department accepts about 30 students to begin the 4-year curriculum every August. The College has appointed 10 full-time faculty members (nine physiotherapists and one exercise scientist). The 10 faculty members include: an associate professor (who is an expatriate), four lecturers, and five assistant lecturers who are new graduates from the first cohort of graduates from the program. Among the four lecturers, one is an expatriate and three are Malawians. Practicing physiotherapists in Malawi who belong to the Physiotherapy Association of Malawi (PAM) have committed themselves to act as part-time lecturers to the program. The Malawi government and the donor community have provided additional lecturers and clinical supervisors.

The Bachelor’s program in Physiotherapy was developed to teach a deep grasp of the pathogenesis and systems of diseases, specialized knowledge and skilled expertise to deliver rehabilitation services, administer assets, and teach lower-cadre rehabilitation workforces. The degree program is a full-time 4-year program. Before students start the first year of the Physiotherapy curriculum, they are enrolled into a 1-year foundation program where they learn premedical sciences such as Mathematics, Biology, Chemistry, Physics, language for communication, and Information Technology as a prerequisite to medical sciences. The first year of the physiotherapy program covers basic medical sciences which are taught in conjunction with other health sciences students by the Divisions of Basic Medical Sciences and Pathology in the COM. From years 2–4, the core subjects of physiotherapy are covered. Clinical work is conducted *via* placements at designated teaching hospitals at district and central hospital levels. The students have hands on experience at selected district hospitals under the supervision of assigned tutors/supervisors. They have regular assignments and a research project. During the fourth year of study, students are exposed to community physiotherapy and also undergo training in management and administration of programs at district and central hospitals. Upon completion of the fourth year of study, students complete a government-paid internship at district and central hospitals. The paid internship ends after 12 months.

## Standards Underlying the Educational Activity

The World Confederation of Physical Therapy recognizes that the education of physical therapists takes places in very diverse cultural and political climates around the world. The WCPT recommends that education for entry level physical therapists be based on university studies of a minimum of 4 years (16), and the baccalaureate degree meets that standard. However, the global trend is to advance from the entry-level physical therapist masters level to doctorate entry qualifications (CAPTE number). This has a significant impact on what is taught and practice expectations around the world (17).

The following resources were used in guiding the curriculum members to participate in a week-long curriculum planning workshop: WCPT Guideline for Physical Therapist Professional Entry Level Education (16); A Normative Model of Physical Therapist Professional Education (18); the curriculum revision which was led by the Consultant from South Africa in 2013; and, course syllabi from the Doctor of Physical Therapy program at California State University, Northridge. As described by the WCPT, the acquisition of broad-based knowledge, critical thinking, self-directed learning and reflection, problem solving, teamwork, and communication is germaine to the practice of physiotherapy (16).

## Rationale for Revision

There were five basic reasons why the original curriculum was revised:

- *to update* outmoded content and methods in light of recent research findings and to correlate the subject matter of the program more closely to current issues and problems in rehabilitation in Malawi;
- *to create* an integrated progression of instruction, with no holes or superfluous content within departmental courses or between cohort levels, that precisely signifies what faculty assess and teach and what undergraduates are supposed to learn from year I to graduation;
- *to improve* awareness across campus of the program’s curriculum and of teaching approaches, priorities, and capabilities among coworkers, as they appear over time in the ongoing college-wide discourse;
- *to document* a printed scope and arrangement of teaching that will aid new faculty members in planning for the teaching obligation when they arrive to the COM, and which will also clarify the program’s curriculum to accreditation teams, prospective students, and others;
- *to deliver* the groundwork for an open-ended dialog every year about what is taught, why it is taught, how it is assessed, and how things might be done otherwise to better help the students and make the most of the COM’s strengths.

## Description of the Process

### Pedagogical Framework

The process of effective curriculum planning and decision-making is the key to the successes of educational programs. The original curriculum for Malawi’s only educational program in Physiotherapy at the University of Malawi’s COM was approved in 2009 for inception of the newly created program in 2010. The revision of the original curriculum was well thought out and has been several years in the planning. The University of Cape Town (South Africa) provided expert advice on course development, revision, and, curricular sequencing in 2013 with help from representatives from the Physiotherapy Associations in Zimbabwe and Malawi as well as from other stakeholders in Malawi. The Head of Department (HOD) from the Physiotherapy Programme made a trip to Singapore and consulted with members of WCPT at the 2015 World Congress. All of these efforts resulted in a revised curriculum; however, the program required capacity building before its implementation. By the time the second cohort had graduated from the program, the program’s faculty had expanded and it was time to attempt to implement the new curriculum in 2016. At that time, there were new faculty members within the department since the original curriculum was approved in 2009 and a curriculum workshop served as local teacher training in curriculum development for these faculty members as well.

### Learning Environment

Groups who had a stake in the outcome of a revised curriculum were invited to a 4-day workshop held at the Department of Physiotherapy in April 2016. Stakeholders included representatives from the Malawi Ministry of Health; the faculty from both the University of Malawi’s COM and Department of Physiotherapy, including the Principal and Dean; members from the PAM, clinical physiotherapy supervisors, alumni (two cohorts had graduated from the program), patients, and, a Fulbright Specialist (19, 20) from the USA. The HOD from Malawi met the Fulbright Specialist at the WCPT Conference in Singapore in 2015 where they both moderated a panel of leaders of physiotherapy international associations. When the HOD returned to Malawi, she contacted the Fulbright officer at the American Embassy in Lilongwe, followed the procedures and submitted a grant proposal detailing the need for curriculum revision and requested that the Fulbright Specialist come to Malawi.

### Objectives

The Curriculum Revision Plan helped guide participants’ development of new and/or revised course curriculum, with the following objectives:

- identify how faculty will integrate their workshop experiences and new knowledge into their course curriculum
- align course revisions with current physiotherapy and university concepts, theories, practices, and policies
- document changes and/or additions faculty intend to make in their courses or teaching practices
- ensure the curriculum contains descriptions of essential elements for effective instruction, including:
  - specific outcomes or skills that students will demonstrate
  - instruction formats to be used
  - instr uctional tools needed
  - assessment plans to determine if students have achieved the set outcomes and skills.

### Pedagogical Format

The research procedures used in this study consisted of systematic document analysis and interviews with educators and the above-mentioned stakeholders. The curriculum workshop was 4 days in length and each 1 of the 4 days of the workshop was devoted to 1 year of the 4-year curriculum: the first day for year I, the second day for Year II, etc. The workshop was an opportunity to come together to find solutions to problems that had cropped up in the course of this new program. The process was structured, and initiated by the consultant, but, then guided and steered by leaders among the stakeholders present.

### Procedures

The stakeholders explored many resources to justify suggested modifications to the originally proposed physiotherapy curriculum in the COM. This process culminated in a week-long Curriculum Workshop to revise the curriculum. The process was structured, and organized/started by the consultant, but, then guided by the leaders within the faculty.

To guarantee that the workshop completely met participants’ educational needs, participants were asked to choose and work on the courses they wished to revise by the conclusion of the workshop. The curriculum was divided into seven course clusters: Basic Medical Sciences, Clinical Sciences, Physiotherapy Sciences, Workplace Practice (including Management), Behavioral Sciences, Research, and Community Physiotherapy.

Each day, stakeholders had the opportunity to make comments and observations on the courses offered in each year of the old curriculum. Then, they were divided into small groups based on course clusters for breakout sessions where as a participant they had selected the courses and objectives they wished to work on and would aid in selecting the course content for the group breakout session work. A fundamental characteristic of the workshop was complete active participation by each attendee: the entire purpose of attendance was to perform and to discover from practical experience.

One of the widespread approaches used in workshops is group conversation on selected topics, the size of the group being small enough to foster complete involvement by each member and big enough for each participant to grow from the knowledge of the others (21). There is nothing special about a small group, but it does extend each member a chance to make his or her own contribution. It gives members the opportunity to discuss and solve the problems of most importance to them. This workshop made everyone (participants and organizers) accountable for assisting to find solutions to the curriculum revision. The breakout sessions were used as working sessions to fill out the college’s curriculum form and write the course syllabus.

At the end of each day, we came together in plenary session for group feedback and consensus on course content. We ended the day with a recap summary session on the day’s activities and the beginning of each new day was a chance for feedback in the opening session on what worked well and what needed improvement from the previous day’s activities. Participants had to act as small group leaders, reporters, or note takers. The consultant was there for consultation and to facilitate where needed (but not to impose solutions). The workshop schedule included plenary sessions, small group discussion and other activities.

The main intent of the curriculum revision plan, which was directed to stakeholders, was to:

- *have an introductory discussion* on learning theories; domains of learning; program’s mission, goals, and objectives; curricular models; course design with skill development; review of how to write instructional goals; and objectives using Bloom’s taxonomy of cognitive learning.
- *discover minimum proficiencies*. What are the essential knowledge data and skills that students must acquire as a result of acceptably finishing a class? What is the purpose of the curriculum?
- *recognize what is in fact taught, in what progression, in each cohort/student*. The objective is for faculty and the academic programs to justly document *when* the content is taught and *what* content is taught. A precise “plan” presents where there are common characteristics, holes, insufficiencies, overemphasized content, etc.
- *measure objectives with the international educational norms to distinguish main concerns and disparities*. This is a vital and rather subjective piece of the method—it obliges faculty members to come to agreement about what they attach importance to with regard to the university’s mission and instructional objectives. This can be contrasted with what international standards deem that students need. And, there is the realization that, usually, it is not possible to lecture on the complete span of international guidelines in every cohort level or course.
- *document the extent and series of the core curriculum*: as soon as the program expressed its objectives for the curriculum, it was vital to chronicle them carefully, for reference and for future deliberations.
- *guide continuing assessment and modification*: Curriculum revision never ends, as requirements, educational standards, faculty, students, and needs change.

## Results

### Immediate Outcomes

The key outcomes of the curriculum revision process included:

- extensive revision of course offerings
- rewritten course learning objectives (written as measurable actions)
- creation of new Teaching/Learning methods in order to achieve new learning objectives
- alignment of student assessment methods with each learning objective
- creation of new courses to allow faculty to teach up-to-date content which match WCPT guidelines (16) and the rehabilitation needs in Malawi
- renumbering of courses as distinguished by course code numbers based on year taught (1, 2, 3, or 4), semester taught (1 or 2), and module taught (#)
- development of a plan for the new curriculum proposal to be reviewed by the University of Malawi Senate (the COM is a constituent college of the University of Malawi) for implementation in August 2017.

### Progress to Date

New courses, course content, and course outlines have been developed and revised and the goal, although ambitious, was to implement the new 4-year curriculum for the next academic year. This timeline has been pushed back 1 year to allow other departments under curriculum review within the COM to be reviewed at the same time as the Department of Physiotherapy by the COM Faculty Senate.

## Discussion

The aim of this article is to describe the application of best practices to bring an outmoded curriculum into alignment with prevailing standards and practices in physical therapy education and practice within the University of Malawi’s COM’s Department of Physiotherapy. In summary, the program’s curriculum was approved in 2009 and a curriculum revision plan was established in 2013. The revised curriculum was not implemented until further capacity building was accomplished within the program. As soon as sufficient capacity building had been achieved, the implementation plan for the new curriculum began with a week-long curriculum workshop in 2016. The process resulted in the establishment of a curricular infrastructure (mission/vision, curricular goals, professional and educational philosophies, and pedagogy). A recap of the curriculum revision plan’s outcomes include a revised/new curriculum, rewritten course learning objectives, alignment of student assessment with learning objectives, new teaching methods, a revised course numbering sequence, and, a plan for approval by the University of Malawi Senate.

### Lessons Learned

The curriculum revision was revitalizing for the faculty. Not many career training occasions are more energizing than meeting with coworkers in a purposeful attempt to discover and discuss how to educate more successfully. The workshop refreshed experts, informed novices, and served to stimulate faculty members.

The challenge was to revise the curriculum in a manner that conformed to the international standards defined by WCPT (16), but, also addressed the needs of Malawi (22). Curricular content needed to be useful for the people of Malawi and requests were made by stakeholders to include content on communicable disease, community-based rehabilitation, and victims of torture from the Dzaleka refugee camp in Malawi with refugees from Burundi, Rwanda, and, the D. R. Congo. It is extremely important for Malawian physiotherapists to have a community-focused approach because most Malawians live in the rural setting (14) and to maintain this focus in the revised curriculum. This process took a dedication among the faculty to think about and reveal their pedagogical methods, establish objectives, and pursue means to meet them. It unified and supported faculty in a manner that, hopefully, over time, will make the educational existence simpler and more satisfying.

Several factors that can restrict curriculum innovation have been identified in the literature. They include issues of time, unavailability of instructional materials, and, instructors’ lack of pedagogy (23). The workshop offered each participant a unique opportunity for uninterrupted time and thought on the curriculum which is universally admitted to be important, but is frequently neglected in all universities.

### Constraints

The faculty in the Department of Physiotherapy must deliver primary content through classroom instruction because of limited access to textbooks and other instructional materials. There is a need to investigate new ways of delivering instruction with the use of distance education (24). In addition, it is important that the faculty are skilled in pedagogy. There is a need to provide pedagogical training to faculty as well as to graduates of the physiotherapy program who are seeking to become faculty at the university level (25). In order for the Physiotherapy program to be sustainable, it will need to produce its own faculty. To that end, two assistant instructors from the first cohort have received grant funding to earn their Masters’ degrees in Physiotherapy from a university in South Africa. Thoughtful faculty with experience in education and physiotherapy will be required for the program to be sustainable and they will need to be engaged in active, relevant research. This approach means that the COM must provide appropriate support for lab-based and community-based research. These resources will support the Physiotherapy program as it prepares the next generation of faculty.

In addition, in order for the Physiotherapy program to be sustainable, graduates will need job availability upon graduation. This will be a challenge, as, to date, graduates from the first two cohorts have not been hired to work in government-run hospitals.

### Next Steps

The next step is to get the revised curriculum approved by the University of Malawi Senate. Curriculum evaluation is an essential phase of curriculum development. Through evaluation a faculty discovers whether a curriculum is fulfilling its purpose and whether students are actually learning. Both summative and formative evaluations of the curriculum have to be considered. In the short term, here is a need to develop a systematic way of obtaining both student and peer assessments of teaching (26). In the long term, assessment of graduate employment needs to be done to provide information about the success in which graduates are being prepared to enter the workforce, including rates of unemployment (27).

The opening of the Physiotherapy Department at the COM has brought great hope for the growth and delivery of physiotherapy services in Malawi. The function and sustainability of the program requires significant planning and partnership to sustain and retain the profession in the future.

### Implications

The curriculum workshop was analytic, from a practical perspective, in serving to recognize holes and redundancies in the program’s curricular continuum. It was emphasized that no one participant was more significant than anyone else. It was further emphasized that the workshop belonged to the participants and would be what they made of it. And, everyone was to keep in mind, that the end of the week was not the end of the workshop, but, rather the start of a long and exciting process which includes challenges posed by implementation of the curriculum, attending to necessary periodic revisions, staff changes, and all other challenges to sustainability. The program’s sustainability will impact the profession of physiotherapy and the society of Malawi. For physiotherapy to flourish in Malawi, it will be necessary to educate the array of stakeholders about the value of this rehabilitation discipline and its impact on quality of life for the individuals and communities it serves.

## Ethics Statement

The work presented in this manuscript did not involve human or animal subjects.

## Author Contributions

Both JB and EC made substantial contributions to the conception or design of the work, drafted the work or revised it critically for important intellectual content, had final approval of the version to be published, and are in agreement to be accountable for all aspects of the work in ensuring that questions related to the accuracy or integrity of any part of the work are appropriately investigated and resolved.

## Conflict of Interest Statement

The authors declare that the research was conducted in the absence of any commercial or financial relationships that could be construed as a potential conflict of interest.
